# Sexual Knowledge, Attitudes, and Motivational Factors Associated with Sexual Activity in Older Adults: A Cross-Sectional Study

**DOI:** 10.3390/ejihpe16060077

**Published:** 2026-05-30

**Authors:** Verónica Estruch-García, María Dolores Gil-Llario, Encarnación Satorres

**Affiliations:** Department of Developmental and Educational Psychology, University of Valencia, 46010 Valencia, Spain; veronica.estruch-garcia@uv.es (V.E.-G.); encarna.satorres@uv.es (E.S.)

**Keywords:** older adults, sexuality, attitudes, motivation, behavior, satisfaction

## Abstract

Background: Sexuality remains an important dimension of well-being in later life, yet it is often surrounded by misconceptions and ageist stereotypes. Understanding sociodemographic, cognitive, attitudinal, motivational, and behavioral aspects of sexuality among older adults may contribute to a more comprehensive view of sexual well-being in aging. Objectives: This study aimed (1) to describe older adults’ knowledge, attitudes, sexual motivation, behaviors, and satisfaction; (2) to examine differences between individuals who reported engaging in partnered eroto-genital activity during the previous six months and those who did not; and (3) to identify sociodemographic (age, gender, religiosity and health condition), cognitive, attitudinal, and motivational factors associated with engagement in partnered eroto-genital activity. Methods: A cross-sectional study was conducted with 186 adults aged 65–88 years (M = 73.29). Participants completed the Aging Sexual Knowledge and Attitudes Scale (ASKAS) and a Sexual Behavior Questionnaire assessing sexual motivation, sexual behaviors, and sexual satisfaction. Descriptive analyses, group comparisons, and hierarchical logistic regression analyses were performed. Results: Participants showed relatively adequate knowledge and generally favorable attitudes, although several misconceptions persisted. Affective behaviors were more frequent than eroto-genital practices. Individuals reporting partnered eroto-genital activity showed higher knowledge, more positive attitudes, greater perceived importance of sexuality, and higher sexual satisfaction. Regression analyses indicated that age, gender, attitudes toward sexuality, and perceived importance of sex were significant predictors of partnered eroto-genital activity, with perceived importance of sex showing the strongest association. Conclusions: Sexuality in later life reflects diverse patterns shaped by cognitive, attitudinal, and especially motivational dimensions. These findings highlight the central role of subjective importance and attitudes in sexual engagement, suggesting that interventions may benefit from moving beyond information provision to also address motivational and relational aspects of sexuality in aging populations.

## 1. Introduction

Sexuality remains a central dimension of well-being across the life span, including in later adulthood, and involves a broad range of experiences such as behaviors, desires, beliefs, attitudes, and interpersonal relationships (World Health Organization, 2006, 2023). However, perceptions of what is considered “sexual” are socially mediated and shaped by biological, psychological, social, economic, and political factors (Doll, 2012; Simpson et al., 2017). Consequently, sexuality may acquire distinct meanings across the life course.

In later adulthood, previous research has suggested that sexual activity is associated with multiple sociodemographic and psychosocial factors. Some studies have reported a progressive decline in certain sexual practices with increasing age (Stentagg et al., 2021; Vasconcelos et al., 2023). This pattern may be associated with biological and health-related changes that become more common during later stages of life, such as chronic health conditions or changes in sexual functioning, which have been associated with lower levels of sexual activity and sexual satisfaction (Gore-Gorszewska et al., 2024; Soysal & Avsar, 2023). However, age may also be associated with generational differences in how sexuality is understood and experienced, given that older individuals have been exposed to different social and cultural contexts, often characterized by more restrictive norms regarding sexual expression and ageist stereotypes that portray aging as an inevitable process of sexual decline and reduced desire (Ayalon & Tesch-Römer, 2018; Syme & Cohn, 2021).

Such stereotypes may lead older adults to perceive themselves as unattractive or asexual, inhibiting sexual expression and fostering the normalization of sexual difficulties as a “natural” consequence of aging (Zhang et al., 2023). In addition, negative perceptions of one’s own aging and health have been associated with lower sexual interest and poorer sexual experiences (Estill et al., 2018), potentially reinforcing withdrawal from sexual activity and discouraging help-seeking behaviors (Sinković & Towler, 2019; Towler et al., 2023).

Gender has also been identified as a relevant factor, with differences observed in sexual activity and sexual experiences between older men and women (Stentagg et al., 2021). Such differences may be related to distinct sexual scripts, social expectations, and life-course trajectories constructed throughout the lifespan (Ayalon et al., 2019; Ricoy-Cano et al., 2020; Saadatmand et al., 2025). In this regard, the previous literature has described the existence of a sexual double standard, according to which women’s sexuality in later life is more likely to be overlooked, stigmatized, or considered less socially acceptable than men’s sexuality (Hinchliff & Gott, 2016). However, gender does not operate in isolation, as gender-related experiences and attitudes may interact with other factors such as age, religiosity, and sociocultural context (Otmani del Barrio et al., 2025). From an intersectional perspective, these overlapping dimensions may contribute to shaping attitudes toward sexuality in later life and provide a broader understanding of the factors influencing sexual experiences and beliefs.

Likewise, sociocultural factors such as religiosity may also play a relevant role in later-life sexuality, as religious beliefs and practices may shape values, norms, and expectations regarding sexual expression and intimate relationships (Hackathorn et al., 2016; Hernandez-Kane & Mahoney, 2018). In this sense, religiosity may influence how individuals perceive sexuality and their attitudes toward different forms of sexual expression.

Moreover, European studies suggest that although some age- and gender-related trends remain relatively stable across countries, important cultural differences exist in the ways sexuality is expressed (López-Sánchez et al., 2023). In Spain, current generations of older adults have experienced important social transformations, including periods characterized by more traditional norms regarding sexuality and stronger religious influences, together with more recent social changes related to sexual rights and affective-sexual diversity (Amigo-Ventureira et al., 2022; López-Sáez et al., 2022).

Although these factors may be associated with sexual experiences during later life, recent research has also challenged ageist stereotypes and highlighted the diversity of ways in which sexuality may be expressed during this stage of life (Ussher et al., 2015). Evidence consistently indicates that many older adults remain sexually active (Boyacıoğlu et al., 2023; Santos-Iglesias et al., 2016), although sexual expression in later life often shifts away from a strictly coitocentric model toward forms of intimacy that emphasize affection, emotional closeness, and physical tenderness (Cameron & Santos-Iglesias, 2024; Ševčíková & Sedláková, 2020). These forms of intimacy frequently include behaviors such as kissing, embracing, and caressing, which may play an important role in maintaining intimacy even when genital sexual activity decreases (Træen et al., 2019). In line with this perspective, Flynn and Gow (2015) suggested that future research should consider distinguishing between different forms of sexual expression, for instance by differentiating between more affective or “light” behaviors (e.g., touching, holding hands, kissing, hugging) and more explicitly eroto-genital practices (e.g., mutual caressing, masturbation, and intercourse) when examining their associations with motivational and relational variables. In this sense, a broader sexual repertoire has been associated with higher levels of sexual satisfaction in later life (Gillespie, 2017).

Therefore, sexual functioning cannot be fully understood solely through biological factors but also requires consideration of beliefs, sexual schemas, and culturally embedded scripts (Nimbi et al., 2022) which have been associated with sexual difficulties, help-seeking behaviors, and treatment adherence (Maxwell et al., 2017; Nimbi et al., 2018, 2019; Tavares et al., 2020). Within this framework, cognitive and attitudinal dimensions such as sexual knowledge and attitudes toward sexuality have been identified as important components associated with sexual motivation, behaviors, and subjective sexual experiences in later life (Cybulski et al., 2018; Even-Zohar & Werner, 2019; Park et al., 2016). These dimensions may be understood as interrelated, such that knowledge, attitudes, motivation, and sexual experiences may coexist and be associated with one another in later life. Greater sexual knowledge may contribute to more favorable beliefs and attitudes regarding sexuality, whereas more positive sexual attitudes have been associated with greater openness toward sexual expression and higher levels of sexual motivation (Carvalho & Nobre, 2011; Das et al., 2012). In turn, sexual motivation has been identified as an important factor associated with sexual experiences and sexual satisfaction in later life (Mark & Lasslo, 2018; Štulhofer et al., 2018). Therefore, these dimensions should not necessarily be considered independent processes but rather interconnected aspects of sexuality that may jointly shape sexual experiences and sexual engagement in later life. The importance of examining sexual knowledge among older adults has been highlighted, particularly in light of increasing rates of sexually transmitted infections among older populations (Fileborn et al., 2018; M. L. Smith et al., 2020), as well as persistent misconceptions about normative sexual changes in aging (Syme & Cohn, 2021).

Consistent with this view, the World Health Organization (2020) has underscored the importance of integrating sexual and reproductive health into national strategies for healthy aging. A growing body of research links active and satisfying sexual lives in older adulthood to improved physical health, psychological well-being, vitality, and overall quality of life (Boyacıoğlu et al., 2023; L. Smith et al., 2019; Vasconcelos et al., 2023; von Humboldt et al., 2025b).

Despite these advances, few studies have simultaneously examined sociodemographic, cognitive, attitudinal, and motivational dimensions of sexuality in later life, particularly in relation to engagement in partnered eroto-genital activity (Wang et al., 2008). Describing sexuality from a broader perspective may provide relevant information for challenging persistent stereotypes that associate aging with an inevitable disappearance of sexual interest or expression by highlighting diverse forms of sexual experience beyond exclusively coital practices. Likewise, comparing cognitive, attitudinal, and motivational characteristics between individuals with and without partnered eroto-genital activity may contribute to identifying patterns associated with different sexual experiences. Finally, simultaneously examining the relative contribution of sociodemographic, cognitive, attitudinal, and motivational factors may contribute to a more comprehensive understanding of the complexity of sexuality and the factors associated with partnered eroto-genital activity in later life.

Accordingly, the present study pursued three main objectives: (1) to describe sexual knowledge, attitudes, motivational factors, sexual behaviors, and sexual satisfaction in older adults; (2) to examine differences across these domains between individuals who reported engaging in partnered eroto-genital behaviors during the previous six months and those who did not; and (3) to identify the relative contribution of sociodemographic (age, gender, religiosity and health condition), cognitive, attitudinal, and motivational factors associated with partnered eroto-genital activity.

Based on previous literature, two hypotheses were proposed. First (H1), individuals reporting partnered eroto-genital activity were expected to show more favorable sexual attitudes, greater sexual knowledge, stronger sexual motivation, and higher sexual satisfaction than those who did not report such activity. Second (H2), sociodemographic (age, gender, religiosity and health condition), cognitive, attitudinal, and motivational factors were expected to be significantly associated with partnered eroto-genital activity.

## 2. Materials and Methods

### 2.1. Participants

Table 1 summarizes the sociodemographic characteristics of the study sample. The study sample comprised 186 participants aged between 65 and 88 years (M = 73.29, SD = 6.35). Participants were recruited through community centers using a convenience sampling approach. Of the participants, 62.4% were women and 37.6% were men, and the vast majority identified as heterosexual (97.3%). Most participants were Spanish (93.5%), with a small proportion from other countries.

Educational attainment ranged from primary education or less (43.8%) to university-level education (20.5%), and most participants reported a middle socioeconomic level (80.6%). Most participants were married or living with a partner (64.5%), while 24.7% were widowed, and the majority lived independently in their own homes (88.2%). Approximately half of the participants identified as non-practicing believers (52.7%), while smaller proportions reported being practicing believers (28.5%) or non-believers/agnostic (18.8%). Given the recruitment strategy, the sample should be considered non-probabilistic.

### 2.2. Measures

Sociodemographic variables. A sociodemographic questionnaire consisting of 12 items was used to collect information on participants’ age, gender (man, woman, or other), sexual orientation (heterosexual, homosexual, bisexual, or asexual), marital status (single, married or with partner, divorced or separated, and widowed), relationship duration, educational level (primary education or less, secondary education, upper secondary education or advanced vocational training, university degree [bachelor’s or master’s level], or doctoral degree), nationality, socioeconomic status (low, middle, or high), religiosity (frequency of religious practice: agnostic, non-practicing believers, or practicing believers), type of housing (residential care, living in their own home, or with family members), and the presence of at least one diagnosed medical condition (Yes/No). Additionally, one item assessed whether participants had access to a private space within their home (Yes/No).

Adapted Version of the Aging Sexual Knowledge and Attitudes Scale (ASKAS). A reduced version of the ASKAS (White, 1982) was used to assess knowledge and attitudes toward sexuality in older adulthood. The instrument comprised two sections: a knowledge section and an attitudes section. The knowledge section included 17 true/false/do not know items assessing understanding of sexuality in later life. Items addressed age-related physiological changes, sexual functioning, sexual satisfaction, perceived attractiveness, beliefs regarding condom use in later life, and common myths and misconceptions surrounding sexuality in older adulthood. Responses were scored by assigning one point to each correct answer and zero points to incorrect or “don’t know” responses. Higher scores reflected greater knowledge of sexuality in later life. The attitudes section consisted of 19 items assessing evaluative judgments regarding sexual expression and sexual rights in later life. Items were rated on a 5-point Likert scale ranging from 1 (strongly disagree) to 5 (strongly agree). Negatively worded items were reverse coded such that higher total scores indicated more permissive and positive attitudes toward sexuality among older adults. Total scores ranged from 19 to 95. The ASKAS is one of the most widely used instruments in research on aging and sexuality (Bauer et al., 2013; Begley et al., 2022; Bouman et al., 2007; Ng & Boey, 2020) and has demonstrated satisfactory psychometric properties (White, 1982, 1998). Subsequent cross-cultural validations have further supported the instrument’s reliability and structural validity (Mahieu et al., 2013; Rodrigues et al., 2024; Viana et al., 2010, 2012; Yan & Lee, 2013). In the current sample, the attitudes subscale demonstrated excellent internal consistency as estimated by McDonald’s omega (ω = 0.974) and the knowledge subscale showed acceptable internal consistency (α = 0.73).

Sexual Behavior Questionnaire. The questionnaire consisted of 14 items developed for this study, based on items used in previous research on sexual activity and function (Avis et al., 2005), with modifications to better capture a broader range of sexual experiences. Core dimensions retained from the original instrument included sexual desire, perceived importance of sex, partnered sexual activity, physical and emotional satisfaction, and frequency of sexual behaviors such as kissing/hugging, sexual touching, oral sex, and intercourse. Some items were modified to better align with the objectives of the present study and characteristics of older adults. Specifically, the original item assessing the importance of sex in life was adapted to assess the perceived importance of sex within the relationship, and sexual intercourse was differentiated into vaginal and anal practices. Additional items assessing sexual interest, desired frequency of sexual activity, and solitary masturbation were incorporated to capture broader aspects of sexuality in later life. It assessed multiple domains of sexual experience using 5-point Likert-type response scales, including sexual interest (e.g., “How would you rate your interest in sex?”, 1 = Very low to 5 = Very high), sexual desire (e.g., “How often have you felt the desire to engage in sexual activity, either alone or with a partner?”, 1 = Never to 5 = Every day), frequency of engagement in different sexual activities (e.g., “On average, how often have you engaged in kissing or hugging?”, 1 = Never to 5 = Every day), and sexual satisfaction (e.g., “How physically pleasurable was your sexual experience?”, 1 = Not at all pleasurable to 5 = Extremely pleasurable). The scale demonstrated high internal consistency, as estimated by McDonald’s omega (ω = 0.89).

### 2.3. Procedure

Data collection began in June 2024 and was completed in December 2025. Several communities’ centers for older adults in Spain were contacted via email, and study information was disseminated to potential participants through the center directors. The study objectives and inclusion criteria (being 65 years or older) were explained to the center directors, who subsequently disseminated the information among potential participants.

Participants were older adults without identified cognitive impairment, as verified by the directors of the participating centers. The participating centers were public community facilities whose main purpose is to provide social and educational spaces where older adults can participate in a variety of activities, including physical exercise programs, handicraft workshops, yoga sessions, and educational talks. These centers function as community settings that promote social participation and active aging.

Individuals who expressed interest were scheduled for an individual face-to-face interview with a member of the research team. These interviews were conducted individually, lasted approximately 30–45 min, and took place in private rooms provided by the centers.

Participants received detailed information about the study and provided written informed consent. Anonymity and confidentiality were guaranteed. No financial or material compensation was provided for participation. The study was approved by the Ethics Committee of Research in Humans of the Ethics Commission in Experimental Research of University of Valencia. All procedures were conducted in accordance with the ethical standards of the Declaration of Helsinki.

### 2.4. Data Analysis

All statistical analyses were conducted using IBM SPSS Statistics (Version 27). Descriptive statistics (means, standard deviations, and percentages) were computed to characterize levels of knowledge, attitudes, sexual motivation and behaviors among adults aged 65 years and older, addressing the first study objective. Sexuality was conceptualized broadly in the present study to capture multiple forms of sexual expression and intimacy in later life, including kissing and hugging, sexual touching, masturbation, oral, vaginal, and anal sexual practices.

To address the second study objective, partnered eroto-genital activity was used as a grouping variable to compare participants who reported partnered eroto-genital activity with those who did not across cognitive, attitudinal, motivational, and satisfaction-related dimensions. Although sexuality was conceptualized broadly, partnered eroto-genital activity was selected for comparative analyses because previous literature suggests that different forms of sexual expression may be associated with distinct relational and experiential characteristics in later life (Flynn & Gow, 2015). Differences between participants reported engaging in partnered eroto-genital activity and those who did not were examined for knowledge and attitudes toward sexuality in older adulthood using independent-samples *t* tests. When the assumption of homogeneity of variances was violated, Welch’s t test was reported. Effect sizes were calculated using Cohen’s d, with values of 0.20, 0.50, and 0.80 interpreted as small, medium, and large effects, respectively. For ordinal variables related to sexual motivation and satisfaction, differences between groups were analyzed using the Mann–Whitney U test. Effect sizes for Mann–Whitney tests were calculated using r (Z/√N). For all statistical tests, statistical significance was established at *p* < 0.05.

Finally, to address the third study objective, partnered eroto-genital activity was treated as the outcome variable in hierarchical logistic regression analyses examining its association with sociodemographic, cognitive, attitudinal, and motivational factors. Predictors were entered using the enter method in three blocks based on previous literature: (1) sociodemographic variables (age, gender, religiosity, and health condition), (2) cognitive variables (knowledge and attitudes toward sexuality in later life), and (3) motivational variables (perceived importance of sex within the relationship). Assumptions for binary logistic regression were assessed prior to the analyses, including multicollinearity, influential observations, model fit, and linearity of the logit. Diagnostic statistics indicated no substantial violations of assumptions. Multicollinearity was low (VIFs = 1.02–1.45), no influential cases were identified (Cook’s distance < 0.39), and model fit was adequate according to the Hosmer–Lemeshow test (*p* = 0.332). Linearity of the logit was generally supported for the continuous predictors.

## 3. Results

### 3.1. Sexual Knowledge, Attitudes and Behavior in Older Adulthood

Participants showed a moderate level of knowledge about sexuality in later life (M = 10.10, SD = 3.08). Most participants showed adequate recognition of some age-related changes; however, several misconceptions were also evident. Notably, 64.5% of participants endorsed the belief that sexual satisfaction inevitably declines after menopause, and 78.5% agreed that older men have greater sexual interest than older women. In addition, 73.1% believed that chronic conditions such as diabetes or hypertension prevent older adults from maintaining a fulfilling sexual life. Misconceptions were also observed regarding condom use in later life. Specifically, 40.9% of participants indicated that sexually active older adults do not need to use condoms. Furthermore, 51.6% attributed the decline in sexual activity in later life primarily to loss of physical attractiveness, and 42.2% failed to recognize that sexual activity may contribute to maintaining sexual responsiveness. Full item-level results are presented in Supplementary Table S1.

Participants reported moderately favorable attitudes toward sexuality in later life (M = 67.75, SD = 12.87), although responses varied depending on the context. A substantial proportion of participants agreed that older adults have little interest in sexuality (41.8%), with slightly higher agreement when referring to widowhood (44.0%).

At the same time, more than half expressed interest in receiving sexual health information (53.3%), and a majority supported sex education programs in residential settings (57.0%) as well as training for institutional staff (63.6%). In addition, most participants endorsed the protection of sexual rights (79.9%) and privacy (69.5%) in institutional contexts. However, more ambivalent attitudes emerged when institutional involvement implied actively facilitating sexual activity, with 27.3% expressing neutral positions and 43.7% showing restrictive attitudes.

Regarding sexual expression, masturbation was considered acceptable by 62.0% of participants for men and 57.6% for women, while 44.0% disapproved of older adults possessing pornographic material. Acceptance of same-sex relationships was relatively high, with 66.2% and 64.5% rejecting their prohibition among women and men, respectively. Full item-level results are presented in Supplementary Table S2.

In addition to cognitive and attitudinal dimensions, variability was observed in motivational aspects of sexuality. Regarding sexual desire, a substantial proportion of participants reported low levels, with 37.4% indicating no sexual desire and 42.3% reporting desire only once or twice per month (see Table 2). A similar pattern was observed for desired frequency of sexual activity, with 33.9% of participants reporting no desired activity and 33.9% indicating a desired frequency of one to two times per month.

With respect to the perceived importance of sex within the relationship, responses were heterogeneous: 19.0% considered it not important at all, 21.5% of little importance, 33.7% moderately important, 20.9% important, and 4.9% very important.

Regarding sexual behavior, affective forms of intimacy were more frequently reported than eroto-genital practices. Kissing and hugging were among the most commonly reported behaviors, with 35.0% indicating daily engagement. In contrast, eroto-genital behaviors were less frequent. Vaginal intercourse was reported as never occurring by 56.2% of participants, while oral and anal sex were reported as absent by 76.3% and 90.9% of the sample, respectively. Solitary masturbation was also relatively infrequent, with 63.4% reporting no engagement in the past six months.

Regarding sexual satisfaction, 9.5% described physical pleasure as extremely pleasurable, 25.2% as very pleasurable, and 32.0% as moderately pleasurable, whereas 11.6% reported it as slightly pleasurable and 21.8% as not at all pleasurable. Emotional pleasure was rated as extremely pleasurable by 19.2% of participants, very pleasurable by 33.6%, and moderately pleasurable by 21.9%, while. 13.7% reported low levels of emotional pleasure and 11.6% indicated no emotional pleasure.

### 3.2. Comparative Analysis Based on Partnered Eroto-Genital Activity

Participants who reported engaging in partnered eroto-genital behaviors during the previous six months (58.1% of the sample) showed significantly higher levels of knowledge about sexuality in later life than those who did not report such behaviors (*p* = 0.004), with a small to-moderate effect size (d = 0.47). Specifically, participants who reported partnered eroto-genital activity obtained higher knowledge scores than those who did not (Table 3).

Significant differences were also observed in attitudes toward sexuality in later life (*p* < 0.001), with a large effect size (d = 0.81). Participants reporting partnered eroto-genital activity expressed substantially more favorable attitudes than those who did not engage in such behaviors.

Beyond cognitive and attitudinal differences, participants reporting partnered eroto-genital activity also showed significantly higher levels of sexual interest (*p* < 0.001, r = 0.37), perceived importance of sex within the relationship (*p* < 0.001, r = 0.33), frequency of sexual desire (*p* < 0.001, r = 0.63), and desired frequency of sexual activity (*p* < 0.001, r = 0.60). Across all variables, participants reporting partnered eroto-genital activity showed higher mean ranks compared with those who did not engage in such behaviors. Notably, the largest differences between groups were observed for motivational aspects of sexuality, particularly sexual desire and desired frequency of sexual activity.

Finally, participants reporting partnered eroto-genital activity also reported significantly higher levels of physical sexual satisfaction than those without such activity (*p* < 0.001, r = 0.41). A similar pattern emerged for emotional sexual satisfaction with higher mean ranks among eroto-genital activity participants than those who did not engage in such behaviors (*p* < 0.001, r = 0.44).

Given these differences, a hierarchical logistic regression analysis was conducted to identify the factors that predict engagement in partnered eroto-genital activity.

### 3.3. Hierarchical Logistic Regression Analysis

The first model including age, gender, religiosity, and health condition was statistically significant (χ^2^(4) = 24.13, *p* < 0.001), explaining 19.6% of the variance (Nagelkerke R^2^ = 0.196), with an overall classification accuracy of 65.2%. Age and gender emerged as significant predictors (B = −0.120, *p*< 0.001; B = −0.887, *p* = 0.027, respectively), indicating that increasing age and being a woman were associated with a lower likelihood of engaging in partnered eroto-genital activity. Religiosity and health condition were not significant predictors (*p* = 0.058 and *p* = 0.382, respectively).

The second model, which added knowledge and attitudes toward sexuality in later life, significantly improved model fit (Δχ^2^(2) = 21.89, *p* < 0.001), increasing explained variance to 34.9% (Nagelkerke R^2^ = 0.349) and classification accuracy to 72.9%. In this model, attitudes toward sexuality were significantly associated with a higher likelihood of sexual activity (B = 0.081; *p* < 0.001), whereas knowledge was not a significant predictor (*p* = 0.490). Age and gender remained significant predictors.

Finally, the inclusion of perceived importance of sex significantly improved the model (Δχ^2^(1) = 9.53, *p* = 0.002). The final model showed a good fit (Hosmer–Lemeshow *p* = 0.332) and a classification accuracy of 76.8%, representing an improvement of 14.9 percentage points compared to the baseline model (61.9%). In this model, perceived importance of sex emerged as a significant positive predictor, while attitudes toward sexuality in later life, age, and gender remained significant (Table 4). Specifically, for each additional year of age, the odds of being sexually active decreased by approximately 11.9% (OR = 0.881), indicating that age remains a robust predictor even when controlling for health and psychosocial variables. Being a woman reduced the odds of sexual activity by 63.6% compared to men (OR = 0.364). Moreover, each additional point in positive attitudes toward sexuality was associated with a 7.9% increase in the likelihood of sexual activity (OR = 1.079). Finally, perceived importance of sex showed the strongest association: for each unit increase, the odds of being sexually active increased by 83.7% (OR = 1.837). Religiosity, health condition, and knowledge were not significantly associated with sexual activity.

## 4. Discussion

The present study addressed three main objectives: to examine older adults’ knowledge, attitudes, motivational aspects, and sexual behaviors in later life; to assess whether engagement in partnered eroto-genital activity was associated with differences across these domains; and to identify the factors associated with such engagement.

In line with the first study objective, the findings suggest a complex and interconnected pattern in which relatively adequate knowledge coexists with persistent misconceptions, moderately favorable attitudes coexist with ambivalence, and sexual behavior reflects both continuity and transformation in later life.

Participants demonstrated partial knowledge of normative physiological changes associated with aging, including alterations in sexual response and the potential impact of medication on desire and performance. However, important misconceptions remained. Notably, more than half of the sample endorsed the belief that women lose the capacity for sexual satisfaction after menopause and that their desire is necessarily lower than that of men. These results are consistent with the persistence of gendered and ageist narratives linking menopause to inevitable sexual decline (Gordillo-Castro et al., 2022). Previous research suggests that women may perceive sexual activity as less relevant after midlife due to bodily changes (Von Humboldt et al., 2025a), which aligns with our results showing that being a woman was associated with a lower likelihood of engaging in partnered eroto-genital activity. Previous research has linked the internalization of decline as normative to lower help-seeking and lower expectations regarding sexual well-being (Masoumi et al., 2025). The present findings suggest that knowledge in later life may be associated not only with access to information but also with internalized sociocultural beliefs related to gender (Åberg et al., 2020).

Regarding health-related knowledge, many participants believed that chronic conditions inevitably compromise sexual life, and a considerable proportion were unaware that continued sexual activity may help maintain sexual responsiveness (Bortz, 2002; Ricoy-Cano et al., 2020; Ševčíková & Sedláková, 2020). Nevertheless, health condition was not significantly associated with sexual activity once other variables were taken into account. At the same time, interpreting sexual difficulties as a natural consequence of aging (Towler et al., 2023) has been associated with lower help-seeking and lower use of adaptive coping strategies (Gocieková et al., 2025; Sinković & Towler, 2019), suggesting a disconnect between beliefs and behavioral outcomes.

In addition, misconceptions regarding sexual risk were also evident (Lyons et al., 2017; Minichiello et al., 2012). Limited awareness of condom use is particularly concerning in light of increasing rates of sexually transmitted infections among older populations (Centers for Disease Control and Prevention, 2024; Giovanna et al., 2019; HIV, STI and Hepatitis B and C Surveillance Unit, 2023). Given that eroto-genital activity was relatively common in our sample, these findings highlight the need for targeted sexual health interventions (Gore-Gorszewska et al., 2025; Granville & Pregler, 2018; World Health Organization, 2020). Encouragingly, participants’ openness to sexual education suggests that such interventions may be well received (Sinković & Towler, 2019).

Attitudes toward sexuality in later life also showed a heterogeneous pattern, indicating that acceptance varies depending on the specific behaviors or contexts under consideration (Cybulski et al., 2018; Sinković & Towler, 2019). Participants expressed strong support for the protection of sexual rights and privacy in institutional settings (Bouman et al., 2006; Villar et al., 2014), yet more ambivalent positions emerged when institutional involvement implied actively facilitating sexual expression, reflecting tensions also documented in previous studies (Fileborn et al., 2015; Gocieková et al., 2025; Mahieu & Gastmans, 2015; Simpson et al., 2017).

Although acceptance of same-sex relationships was generally high, the presence of neutral responses suggests that attitudes toward sexual diversity in later life may still be evolving (Fredriksen-Goldsen & Muraco, 2010; Hughes & King, 2018). Given the age range of the present sample, many participants experienced important social and historical transitions in Spain. These transitions included periods characterized by more traditional norms regarding sexuality, gender roles, and stronger religious influences in social life. Together with individual factors such as religiosity, these experiences may help explain the coexistence of both more permissive and more restrictive attitudes toward sexual diversity.

At the same time, a substantial proportion of participants endorsed the belief that older adults have little interest in sexuality. This perception, consistent with ageist stereotypes portraying older adults as asexual (Sinković & Towler, 2019; Syme & Cohn, 2021), may be associated with the normalization of reduced sexual expression (Fileborn et al., 2015). However, this belief coexisted with clear evidence of ongoing sexual interest and activity, as a considerable proportion of participants remained sexually active (Cismaru-Inescu et al., 2022; Fischer et al., 2021; Freak-Poli et al., 2017; Schick et al., 2010).

In line with our first hypothesis, participants reporting partnered eroto-genital activity showed more favorable attitudes toward sexuality, greater knowledge regarding sexuality in later life, and higher levels of sexual motivation than those who did not report such activity. In addition, individuals reporting partnered eroto-genital activity also presented higher levels of emotional and physical sexual satisfaction. These findings suggest that sexual experiences in later life may involve not only behavioral differences but also broader cognitive, attitudinal, and motivational dimensions.

This pattern is consistent with research based on the Aging Sexual Knowledge and Attitudes Scale (ASKAS; White, 1982), which identifies attitudes as key correlates of sexual expression, satisfaction, and well-being (Cybulski et al., 2018; Park et al., 2016; Wang et al., 2008). These findings can be interpreted in light of internalized stereotypes that have been associated with sexual self-perceptions and behaviors (Sinković & Towler, 2019; Syme & Cohn, 2021), whereas beliefs framing sexuality as normative and beneficial in later life have been associated with greater well-being and sexual engagement (Fischer et al., 2018; Graf & Patrick, 2014).

Beyond attitudinal factors, the perceived importance of sex within the relationship emerged as the strongest predictor of partnered eroto-genital activity. This finding aligns with previous research highlighting the role of sexual motivation and relational meaning in later-life sexuality (Flynn & Gow, 2015; Penhollow et al., 2009). Sexual experiences may be perceived not only as physical experiences themselves but also as a means of maintaining intimacy and emotional closeness within relationships. Therefore, the subjective importance attributed to sexuality may capture relational and motivational dimensions that are closely associated with sexual experiences in later life and may help explain its stronger association compared with cognitive dimensions alone. However, this finding should be interpreted with some caution because the perceived importance of sex and sexual activity, although conceptually distinct, may represent closely related dimensions.

Previous studies have described discrepancies between sexual desire and sexual behavior (Beier et al., 2020). In the present study, this pattern may be associated with contextual factors such as partner availability, relationship dynamics, or differences in the social acceptability of specific practices (Portellos et al., 2023). In our study, for instance, masturbation was reported less frequently than partnered sexual activity, which may be related to its lower social acceptance and to the ambivalence observed toward these practices (Cameron & Santos-Iglesias, 2024; Ševčíková & Sedláková, 2020).

Consistent with this, although affective forms of intimacy such as kissing and hugging were more frequently reported than eroto-genital practices, the latter remained present within partnered relationships (Waite et al., 2009; Tetley et al., 2018). This suggests that sexuality in later life may be expressed through different combinations of affective and genital experiences. Interestingly, although affective expressions of intimacy appeared more frequent, emotional and physical satisfaction were higher among individuals reporting partnered eroto-genital activity (Cismaru-Inescu et al., 2022; Heiman et al., 2011; Træen et al., 2017). This pattern may suggest that frequency alone does not necessarily correspond to the subjective significance of specific sexual experiences. While affective expressions may represent important aspects of everyday intimacy, partnered eroto-genital experiences may remain associated with aspects of sexuality perceived as particularly meaningful or satisfying (Erens et al., 2019; Ševčíková & Sedláková, 2020). Rather than representing alternative forms of sexuality, affective and eroto-genital expressions may constitute complementary dimensions contributing differently to sexual well-being in later life.

In line with our second hypothesis, sociodemographic variables also contributed to explaining sexual activity patterns. Age and gender remained significant predictors even after controlling for cognitive, attitudinal, and motivational variables (Cremé-Lobaina et al., 2017), whereas, contrary to expectations, health condition and religiosity did not.

Older adults and their partners may develop compensatory strategies, including alternative forms of intimacy and sexual expression, helping maintain sexual experiences despite health-related limitations (Erens et al., 2019). Previous research has reported mixed findings regarding these variables (Sinković & Towler, 2019; Syme & Cohn, 2021), and the present results suggest that their influence may become less direct when psychological and motivational dimensions are considered simultaneously.

Regarding the role of age, Miller et al. (2021) highlight that aging encompasses not only physiological changes but also psychological, social, and contextual experiences accumulated throughout the life course. Therefore, although age may be associated with changes in sexual response and sexual functioning, it may also capture generational influences. Current generations of older adults were socialized within contexts where sexuality was often perceived as a taboo topic, potentially shaping beliefs and expectations regarding sexuality in later life.

Likewise, gender differences in sexuality may not exclusively reflect biological factors. Previous literature suggests that women and men are exposed to different sexual scripts and social expectations throughout life (Ayalon et al., 2019; Ricoy-Cano et al., 2020; Saadatmand et al., 2025). Sexual activity in men has often been socially associated with successful aging and masculinity, whereas women’s sexuality has historically been more restricted to reproductive and caregiving roles and more frequently invisibilized in later life (Hinchliff & Gott, 2016). This may be particularly relevant within the Spanish context, where current generations of older adults grew up within environments characterized by more traditional gender norms and unequal expectations regarding sexuality and intimate relationships.

Although religiosity was not significant in the present model, the previous literature suggests that religious beliefs may influence sexual attitudes indirectly through values and norms regarding sexuality (Sinković & Towler, 2019). These results may be explained by the fact that the interaction between religiosity and marital status was not taken into account (Hackathorn et al., 2016; Hernandez-Kane & Mahoney, 2018). This issue should be explored in greater depth in future studies.

Taken together, these findings support a biopsychosocial perspective (Nimbi et al., 2022), suggesting that sexuality in later life should not be understood through isolated variables, but through the interrelationships among knowledge, attitudes, motivational dimensions, and broader sociocultural experiences. Consistent with an intersectional perspective, these findings also highlight the importance of considering how sociodemographic factors, such as gender, age, religiosity, and sociocultural context, may contribute to understanding sexuality in later life (Otmani del Barrio et al., 2025). In this way, the results contribute to challenging simplistic narratives that portray later-life sexuality either as a linear decline or as a mere continuation of earlier life stages (Fileborn et al., 2017; Gore-Gorszewska et al., 2025).

### Limitations

Several limitations should be considered when interpreting these findings. First, the cross-sectional design of the study precludes causal inferences regarding the relationships between sociodemographic variables, sexual knowledge, attitudes, motivation, and sexual behaviors. Future longitudinal research is needed to clarify the temporal ordering of these variables and to explore potential bidirectional relationships between sexual engagement and psychological dimensions of sexuality in later life.

Second, participation was voluntary and focused on a sensitive topic, which may have resulted in self-selection bias. Individuals who chose to participate might have been more open to discussing sexuality or more interested in the topic, potentially leading to an over representation of more favorable attitudes toward sexuality.

Third, participants were recruited using a convenience sampling strategy, primarily through community centers for older adults. Although participants represented community-dwelling older adults, recruitment through these settings may have resulted in a sample characterized by higher levels of social participation and engagement in active aging activities. These characteristics may be associated with differences in attitudes, motivations, or experiences related to sexuality and should therefore be considered when interpreting the findings.

Finally, contextual characteristics of the recruitment settings (e.g., rural versus urban environments) were not systematically collected. Given that sociocultural contexts may influence sexual attitudes and experiences in later life, future studies should further examine the role of these contextual factors.

## 5. Conclusions

Importantly, the results suggest that while knowledge contributes to describing sexual beliefs and differentiating between individuals, its explanatory role becomes limited when attitudinal and motivational factors are considered simultaneously. In this regard, attitudes toward sexuality in later life and, particularly, the perceived importance of sex within the relationship emerged as more central factors associated with sexual engagement. These findings underscore the multidimensional nature of sexual well-being in aging, suggesting that sexuality in later life is not solely determined by biological or health-related factors, but is strongly shaped by psychological and relational dimensions. As in younger populations, overly generalized assumptions about older adults’ sexuality may obscure the diversity of experiences that characterize this stage of life. In line with previous research, these results support the need to normalize diverse forms of sexual expression and to integrate sexual health into broader strategies promoting healthy and active aging (Fileborn et al., 2017).

## Figures and Tables

**Table 1:** Sociodemographic characteristics.

Variable		(%)
Gender	Men	37.6
	Women	62.4
	Other	0
Sexual orientation	Heterosexual	97.3
	Homosexual	1.1
	Bisexual	1.6
	Asexual	0
Nationality	Spanish	93.5
	Romanian	1.6
	Chilean	1.6
	Morocco	1.1
	Portuguese	1.1
	Colombian	0.6
	Bolivian	0.5
Education level	Primary education or less	43.8
	Secondary education	18.9
	Upper secondary education/vocational training	16.8
	University degree (bachelor’s or master’s level)	18.9
	Doctoral degree	1.6
Marital status	Single	3.8
	Married or in a relationship	64.5
	Separated/Divorced	7.0
	Widowed	24.7
Living arrangements	Own home	88.2
	Residential care	6.4
	Family members‘ home	5.4
Religiosity	Agnostic	18.8
	Non-practicing believers	52.7
	Practicing believers	28.5
Health condition	Yes	70.4
	No	29.6

**Table 2:** Frequency distribution of sexual desire, desired sexual activity, and sexual behaviors in the past six months.

	Never (%)	1–2 Times per Month (%)	Once per Week (%)	More than Once per Week (%)	Daily (%)
Sexual desire	37.4	42.3	7.1	11.0	2.2
Desired frequency of sexual activity	33.9	33.9	20.8	9.8	1.6
Solitary Masturbation (%)	63.4	22.5	7.9	5.6	0.6
Kissing or Hugging (%)	21.1	13.9	16.1	13.9	35.0
Sexual Touching (%)	43.8	24.7	15.2	12.4	3.9
Vaginal Intercourse (%)	56.2	28.1	11.2	4.5	0.0
Oral Sex (%)	76.3	13.0	6.8	2.8	1.1
Anal Intercourse (%)	90.9	6.8	0.6	0.6	1.1

**Table 3:** Differences in sexual knowledge, attitudes, and motivational variables according to partnered eroto-genital activity.

Variable	No Activity M (SD)/MR	Activity M (SD)/MR	Test Statistic	*p*	Effect Size
Sexual knowledge in later life	9.28 (3.47)	10.70 (2.66)	t(131.18) = −2.94	0.004	d = 0.47
Attitudes toward sexuality in later life	62.23 (12.94)	71.88 (11.10)	t(172) = −5.29	<0.001	d = 0.81
Sexual interest	68.13	105.77	U = 2260.00	<0.001	r = 0.37
Importance of sex	61.86	93.05	U = 1881.50	<0.001	r = 0.33
Sexual desire	54.45	115.63	U = 1234.00	<0.001	r = 0.63
Desired frequency	55.27	115.04	U = 1295.50	<0.001	r = 0.60
Physical satisfaction	46.76	83.70	U = 1061.00	<0.001	r = 0.41
Emotional satisfaction	44.33	83.71	U = 956.50	<0.001	r = 0.44

**Table 4:** Predictors of partnered eroto-genital activity: Results of hierarchical logistic regression analysis.

Predictor	B	SE	Wald	*p*	OR	95% CI OR
Age	−0.127	0.038	10.89	0.001	0.881	[0.817, 0.950]
Gender (woman)	−1.012	0.462	4.80	0.028	0.364	[0.147, 0.899]
Religiosity	−0.166	0.327	0.26	0.612	0.847	[0.446, 1.608]
Health condition	0.709	0.443	2.56	0.110	2.032	[0.852, 4.846]
Attitudes toward sexuality in later life	0.076	0.022	12.39	<0.001	1.079	[1.034, 1.125]
Sexual knowledge in later life	−0.122	0.085	2.09	0.148	0.885	[0.750, 1.044]
Importance of sex	0.608	0.205	8.78	0.003	1.837	[1.228, 2.747]

Note. OR = odds ratio; CI = confidence interval. Gender coded as 0 = man, 1 = woman. Health condition coded as 0 = no diagnosed condition, 1 = diagnosed condition.

## Data Availability

The data are not publicly available due to privacy or ethical restrictions. Research data are not shared.

## Supplementary Materials

The following supporting information can be downloaded at: https://www.mdpi.com/article/10.3390/ejihpe16060077/s1, Table S1: Percentage of correct and incorrect responses to knowledge items on sexuality in older adulthood; Table S2: Percentage distribution of responses to attitude items on sexuality in older adulthood.

## References

[B1-ejihpe-16-00077] Amigo-Ventureira A., DePalma R., Durán-Bouza M. (2022). Homophobia and transphobia among Spanish practicing and future teachers. American Journal of Sexuality Education.

[B2-ejihpe-16-00077] Avis N. E., Zhao X., Johannes C. B., Ory M., Brockwell S., Greendale G. A. (2005). Correlates of sexual function among multi-ethnic middle-aged women: Results from the Study of Women’s Health Across the Nation (SWAN). Menopause.

[B3-ejihpe-16-00077] Ayalon L., Gewirtz-Meydan A., Levkovich I. (2019). Older adults’ coping strategies with changes in sexual functioning: Results from qualitative research. The Journal of Sexual Medicine.

[B4-ejihpe-16-00077] Ayalon L., Tesch-Römer C. (2018). Contemporary perspectives on ageism.

[B5-ejihpe-16-00077] Åberg E., Kukkonen I., Sarpila O. (2020). From double to triple standards of ageing: Perceptions of physical appearance at the intersections of age, gender, and class. Journal of Aging Studies.

[B6-ejihpe-16-00077] Bauer M., McAuliffe L., Nay R., Chenco C. (2013). Sexuality in older adults: Effect of an education intervention on attitudes and beliefs of residential aged care staff. Educational Gerontology.

[B7-ejihpe-16-00077] Begley T., Daly L., Downes C., de Vries J., Sharek D., Higgins A. (2022). Training programmes for practitioners in sexual health promotion: An integrative literature review of evaluations. Sex Education.

[B8-ejihpe-16-00077] Beier K.-M., Kossow S., Zhou Y., Kroll F., Neumann B., Konrad A., Mundt I., Steinhagen-Thiessen E., Demuth I., Rosada A., Pauls A. (2020). The gap between desires and reality in sexuality of males and females aged 60 and over: Results from the berlin aging study II (base-II). Sexologies.

[B9-ejihpe-16-00077] Bortz W. M. (2002). A conceptual framework of frailty: A review. The Journals of Gerontology: Series A.

[B10-ejihpe-16-00077] Bouman W. P., Arcelus J., Benbow S. M. (2006). Nottingham study of sexuality & ageing (NoSSA I). Attitudes regarding sexuality and older people: A review of the literature. Sexual and Relationship Therapy.

[B11-ejihpe-16-00077] Bouman W. P., Arcelus J., Benbow S. M. (2007). Nottingham study of sexuality and ageing (NoSSA II). Attitudes of care staff regarding sexuality and residents: A study in residential and nursing homes. Sexual and Relationship Therapy.

[B12-ejihpe-16-00077] Boyacıoğlu N. E., Oflaz F., Karaahmet A. Y., Hodaeı B. K., Afşin Y., Erpolat Taşabat S. (2023). Sexuality, quality of life and psychological well-being in older adults: A correlational study. European Journal of Obstetrics & Gynecology and Reproductive Biology: X.

[B13-ejihpe-16-00077] Cameron J., Santos-Iglesias P. (2024). Sexual activity of older adults: A systematic review of the literature. International Journal of Sexual Health.

[B14-ejihpe-16-00077] Carvalho J., Nobre P. (2011). Predictors of men’s sexual desire: The role of psychological, cognitive-emotional, relational, and medical factors. The Journal of Sex Research.

[B15-ejihpe-16-00077] Centers for Disease Control and Prevention (2024). NCHHSTP AtlasPlus.

[B16-ejihpe-16-00077] Cismaru-Inescu A., Hahaut B., Adam S., Nobels A., Beaulieu M., Vandeviver C., Keygnaert I., Nisen L. (2022). Sexual activity and physical tenderness in older adults: Prevalence and associated characteristics from a Belgian study. The Journal of Sexual Medicine.

[B17-ejihpe-16-00077] Cremé-Lobaina E., Alvarez-Cortés J. T., Pérez-Hechavarría G. A., Fernández-González P., Riveaux-Verdecia R. (2017). Sexual health in elderly from a family doctor’s office. MEDISAN.

[B18-ejihpe-16-00077] Cybulski M., Cybulski L., Krajewska-Kulak E., Orzechowska M., Cwalina U., Jasinski M. (2018). Sexual quality of life, sexual knowledge, and attitudes of older adults on the example of inhabitants over 60s of Bialystok, Poland. Frontiers in Psychology.

[B19-ejihpe-16-00077] Das A., Waite L., Laumann E. O. (2012). Sexual expression over the life course. Sex for life: From virginity to Viagra, how sexuality changes throughout our lives.

[B20-ejihpe-16-00077] Doll G. A. (2012). Sexuality and long-term care: Understanding and supporting the needs of older adults.

[B21-ejihpe-16-00077] Erens B., Mitchell K. R., Gibson L., Datta J., Lewis R., Field N., Wellings K. (2019). Health status, sexual activity and satisfaction among older people in Britain: A mixed methods study. PLoS ONE.

[B22-ejihpe-16-00077] Estill A., Mock S. E., Schryer E., Eibach R. P. (2018). The effects of subjective age and aging attitudes on mid- to late-life sexuality. The Journal of Sex Research.

[B23-ejihpe-16-00077] Even-Zohar A., Werner S. (2019). Older adults and sexuality in Israel: Knowledge, attitudes, sexual activity and quality of life. Journal of Aging Science.

[B24-ejihpe-16-00077] Fileborn B., Brown G., Lyons A., Hinchliff S., Heywood W., Minichiello V., Malta S., Barrett C., Crameri P. (2018). Safer sex in later life: Qualitative interviews with older Australians on their understandings and practices of safer sex. The Journal of Sex Research.

[B25-ejihpe-16-00077] Fileborn B., Lyons A., Hinchliff S., Brown G., Heywood W., Dow B., Malta S., Minichiello V. (2017). Improving the sexual lives of older Australians: Perspectives from a qualitative study. Australasian Journal on Ageing.

[B26-ejihpe-16-00077] Fileborn B., Thorpe R., Haekes G., Minichiello V., Pitts M. (2015). Sex and the (older) single girl: Experiences of sex and dating in later life. Journal of Aging Studies.

[B27-ejihpe-16-00077] Fischer N., Štulhofer A., Hald G. M., Carvalheira A., Træen B. (2021). Sexual satisfaction in older heterosexual couples from four European countries: Exploring the roles of actual and perceived discrepancy in sexual interest. Journal of Sex Research.

[B28-ejihpe-16-00077] Fischer N., Træen B., Hald G. M. (2018). Predicting partnered sexual activity among older adults in four European countries: The role of attitudes, health, and relationship factors. Sexual and Relationship Therapy.

[B29-ejihpe-16-00077] Flynn T. J., Gow A. J. (2015). Examining associations between sexual behaviours and quality of life in older adults. Age and Ageing.

[B30-ejihpe-16-00077] Freak-Poli R., Kirkman M., De Castro Lima G., Direk N., Franco O. H., Tiemeier H. (2017). Sexual activity and physical tenderness in older adults: Cross-sectional prevalence and associated characteristics. The Journal of Sexual Medicine.

[B31-ejihpe-16-00077] Fredriksen-Goldsen K. I., Muraco A. (2010). Aging and sexual orientation: A 25-year review of the literature. Research on Aging.

[B32-ejihpe-16-00077] Gillespie B. J. (2017). Correlates of sex frequency and sexual satisfaction among partnered older adults. Journal of Sex & Marital Therapy.

[B33-ejihpe-16-00077] Giovanna G. N., Gaudenci E. M., da Silveira R. E., Alves L. A., Sousa B. S., da Silva A. (2019). Knowledge about HIV/AIDS in older adults using the services of Family Health Strategy. Journal of the Brazilian Society of Tropical Medicine.

[B34-ejihpe-16-00077] Gocieková V., Stašek A., Ševčíková A., Gore-Gorszewska G. (2025). The role of ageist sexual stereotypes in the network of sexual difficulties, sex and relationship satisfaction among adults aged 50+. The Journal of Sex Research.

[B35-ejihpe-16-00077] Gordillo-Castro A., López-Márquez G., González T., Vallejo-Arce M. (2022). Attitudes and perceptions of older adults about their sexuality. MASKANA.

[B36-ejihpe-16-00077] Gore-Gorszewska G., Ševčíková A., Bártová K., Krejčová L., Kalenská L., Androvičová R., Weiss P., Klapilová K. (2025). Silent struggles: Help-seeking barriers for sexual difficulties among adults 50+ in Czechia. Frontiers in Psychology.

[B37-ejihpe-16-00077] Gore-Gorszewska G., Ševčíková A., Gottfried J. (2024). Predicting changes to sexual activity in later life: A longitudinal study. Sexuality Research and Social Policy.

[B38-ejihpe-16-00077] Graf A. S., Patrick J. H. (2014). The influence of sexual attitudes on mid- to late-life sexual well-being: Age, not gender, as a salient factor. The International Journal of Aging and Human Development.

[B39-ejihpe-16-00077] Granville L., Pregler J. (2018). Women’s sexual health and aging. Journal of the American Geriatrics Society.

[B40-ejihpe-16-00077] Hackathorn J. M., Ashdown B. K., Rife S. C. (2016). The sacred bed: Sex guilt mediates religiosity and satisfaction for unmarried people. Sexuality & Culture.

[B41-ejihpe-16-00077] Heiman J. R., Long J. S., Smith S. N., Fisher W. A., Sand M. S., Rosen R. C. (2011). Sexual satisfaction and relationship happiness in midlife and older couples in five countries. Archives of Sexual Behavior.

[B42-ejihpe-16-00077] Hernandez-Kane K. M., Mahoney A. (2018). Sex through a sacred lens: Longitudinal effects of sanctification of marital sexuality. Journal of Family Psychology.

[B43-ejihpe-16-00077] Hinchliff S., Gott M., Peel E., Harding R., Westwood S. (2016). Ageing and sexuality in Western societies: Changing perspectives on sexual activity, sexual expression, and the sexy older body. Ageing and sexualities: Interdisciplinary perspectives.

[B44-ejihpe-16-00077] HIV, STI and Hepatitis B and C Surveillance Unit (2023). Vigilancia epidemiológica de las infecciones de transmisión sexual *[Epidemiological surveillance of sexually transmitted infections]*.

[B45-ejihpe-16-00077] Hughes M., King A. (2018). Representations of LGBT ageing and older people in Australia and the UK. Journal of Sociology.

[B46-ejihpe-16-00077] López-Sáez M. A., García-Dauder D., Montero I., Lecuona O. (2022). Adaptation and validation of the evasive attitudes of sexual orientation scale into Spanish. Journal of Homosexuality.

[B47-ejihpe-16-00077] López-Sánchez G. F., Oliva-Lozano J. M., Muyor J. M., Smith L., Smith L., Grabovac I. (2023). Levels and trends of sexual activity in older adults. Sexual behaviour and health in older adults.

[B48-ejihpe-16-00077] Lyons A., Heywood W., Fileborn B., Minichiello V., Barrett C., Brown G., Hinchliff S., Malta S., Crameri P. (2017). Sexually active older Australian’s knowledge of sexually transmitted infections and safer sexual practices. Australian and New Zealand Journal of Public Health.

[B49-ejihpe-16-00077] Mahieu L., de Casterlé B. D., Van Elssen K., Gastmans C. (2013). Nurses’ knowledge and attitudes towards aged sexuality: Validity and internal consistency of the Dutch version of the Aging Sexual Knowledge and Attitudes Scale. Journal of Advanced Nursing.

[B50-ejihpe-16-00077] Mahieu L., Gastmans C. (2015). Older residents’ perspectives on aged sexuality in institutionalized elderly care: A systematic literature review. International Journal of Nursing Studies.

[B51-ejihpe-16-00077] Mark K. P., Lasslo J. A. (2018). Maintaining sexual desire in long-term relationships: A systematic review and conceptual model. The Journal of Sex Research.

[B52-ejihpe-16-00077] Masoumi M., Keramat A., Farjamfar M., Talebi S. S. (2025). Sexual health needs of postmenopausal women: A systematic review. Journal of Sex & Marital Therapy.

[B53-ejihpe-16-00077] Maxwell J. A., Muise A., MacDonald G., Day L. C., Rosen N. O., Impett E. A. (2017). How implicit theories of sexuality shape sexual and relationship well-being. Journal of Personality and Social Psychology.

[B54-ejihpe-16-00077] Miller L. R., García J. R., Gesselman A. (2021). Dating and sexualities across the life course: The interactive effects of aging and gender. Journal of Aging Studies.

[B55-ejihpe-16-00077] Minichiello V., Rahman S., Hawkes G., Pitts M. (2012). STI epidemiology in the global older population: Emerging challenges. Perspectives in Public Health.

[B56-ejihpe-16-00077] Ng A. H. N., Boey K. W. (2020). Enhancement of Hong Kong nursing students’ knowledge and attitudes regarding sexuality in older adults. International Journal of Sexual Health.

[B57-ejihpe-16-00077] Nimbi F. M., Galizia R., Rossi R., Limoncin E., Giacomo C., Fontanesi L., Jannini E. A., Simonello C., Tambelli R. (2022). The biopsychosocial model and the sex-positive approach: An integrative perspective for sexology and general health care. Sexuality Research and Social Policy.

[B58-ejihpe-16-00077] Nimbi F. M., Rossi R., Tripodi F., Wylie K., Simonelli C. (2019). A biopsychosocial model for the counseling of hormonal contraceptives: A review of the psychological, relational, sexual, and cultural elements involved in the choice of contraceptive method. Sexual Medicine Reviews.

[B59-ejihpe-16-00077] Nimbi F. M., Tripodi F., Rossi R., Simonelli C. (2018). Testing a conceptual model for men’s sexual desire referring to automatic thoughts, emotions, sexual function, and sexism. The Journal of Sexual Medicine.

[B60-ejihpe-16-00077] Otmani del Barrio M., del Pilar-Labarda M., Awor P., Echvarria M. I., Mier A. R., Peeling R. W., Chamas C., Reeder J. C. (2025). Why does an intersectional gender approach matter for social innovations in health?. BMJ Innovations.

[B61-ejihpe-16-00077] Park H. K., Kang S. K., Park S. (2016). Sexual knowledge, sexual attitude, and life satisfaction among Korean older adults: Implications for educational programs. Sexuality & Disability.

[B62-ejihpe-16-00077] Penhollow T. M., Young M., Denny G. (2009). Predictors of quality of life, sexual intercourse, and sexual satisfaction among active older adults. American Journal of Health Education.

[B63-ejihpe-16-00077] Portellos A., Lynch C., Joosten A. (2023). Sexuality and ageing: A mixed methods explorative study of older adult’s experiences, attitudes, and support needs. The British Journal of Occupational Therapy.

[B64-ejihpe-16-00077] Ricoy-Cano A. J., Obrero-Gaitán E., Caravaca-Sánchez F., Fuente-Robles Y. M. D. L. (2020). Factors conditioning sexual behavior in older adults: A systematic review of qualitative studies. Journal of Clinical Medicine.

[B65-ejihpe-16-00077] Rodrigues L., Peixoto M. M., Patrão A. L., Santos L., Magalhães S. I., Nogueira C. (2024). Psychometric properties and gender invariance of the Portuguese (European) version of the Aging Sexual Knowledge and Attitudes Scale. Journal of Cross-Cultural Gerontology.

[B66-ejihpe-16-00077] Saadatmand F., Beigi M., Heidari Z., Savabi-Esfahani M. (2025). Perceived sexual health needs of older women: A systematic review. Iranian Journal of Nursing and Midwifery Research.

[B67-ejihpe-16-00077] Santos-Iglesias P., Byers E. S., Moglia R. (2016). Sexual well-being of older men and women. The Canadian Journal of Human Sexuality.

[B68-ejihpe-16-00077] Schick V., Herbenick D., Reece M., Sanders S. A., Dodge B., Middlestadt S. E., Fortenberry J. D. (2010). Sexual behaviors, condom use, and sexual health of Americans over 50: Implications for sexual health promotion for older adults. The Journal of Sexual Medicine.

[B69-ejihpe-16-00077] Simpson P., Horne M., Brown L. J., Wilson C. B., Dickinson T., Torkington K. (2017). Old(er) care home residents and sexual/intimate citizenship. Ageing & Society.

[B70-ejihpe-16-00077] Sinković M., Towler L. (2019). Sexual aging: A systematic review of qualitative research on the sexuality and sexual health of older adults. Qualitative Health Research.

[B71-ejihpe-16-00077] Smith L., Yang L., Veronese N., Soysal P., Stubbs B., Jackson S. E. (2019). Sexual Activity is Associated with Greater Enjoyment of Life in Older Adults. Sexual Medicine.

[B72-ejihpe-16-00077] Smith M. L., Bergeron C. D., Goltz H. H., Coffey T., Boolani A. (2020). Sexually transmitted infection knowledge among older adults: Psychometrics and test–retest reliability. International Journal of Environmental Research and Public Health.

[B73-ejihpe-16-00077] Soysal P., Avsar E. (2023). Sexual activity and physical health benefits in older adults. Sexual behaviour and health in older adults.

[B74-ejihpe-16-00077] Stentagg M., Skär L., Berglund J. S., Lindberg T. (2021). Cross-sectional study of sexual activity and satisfaction among older adult’s ≥60 years of age. Sexual Medicine.

[B75-ejihpe-16-00077] Syme M. L., Cohn T. J. (2021). Aging sexual stereotypes and sexual expression in mid- and later life: Examining the stereotype matching effect. Aging & Mental Health.

[B76-ejihpe-16-00077] Ševčíková A., Sedláková T. (2020). The role of sexual activity from the perspective of older adults: A qualitative study. Archives of Sexual Behavior.

[B77-ejihpe-16-00077] Štulhofer A., Hinchliff S., Jurin T., Carvalheira A., Træen B. (2018). Successful aging, change in sexual interest and sexual satisfaction in couples from four European countries. European Journal of Ageing.

[B78-ejihpe-16-00077] Tavares I. M., Moura C. V., Nobre P. J. (2020). The role of cognitive processing factors in sexual function and dysfunction in women and men: A systematic review. Sexual Medicine Reviews.

[B79-ejihpe-16-00077] Tetley J., Lee D. M., Nazroo J., Hinchliff S. (2018). Let’s talk about sex—What do older men and women say about their sexual relations and sexual activities? A qualitative analysis of ELSA Wave 6 data. Ageing and Society.

[B80-ejihpe-16-00077] Towler L. B., Graham C. A., Bishop F. L., Hinchliff S. (2023). Sex and relationships in later life: Older adults’ experiences and perceptions of sexual changes. The Journal of Sex Research.

[B81-ejihpe-16-00077] Træen B., Hald G. M., Graham C. A., Enzlin P., Janssen E., Kvalem I. L., Carvalheira A., Štulhofer A. (2017). Sexuality in older adults (65+)—An overview of the literature, part 1: Sexual function and its difficulties. International Journal of Sexual Health.

[B82-ejihpe-16-00077] Træen B., Štulhofer A., Janssen E., Carvalheira A. A., Hald G. M., Lange T., Graham C. (2019). Sexual activity and sexual satisfaction among older adults in four European countries. Archives of Sexual Behavior.

[B83-ejihpe-16-00077] Ussher J. M., Perz J., Parton C., McHugh M. C., Chrisler J. C. (2015). Menopause and sexuality: Resisting representations of the abject asexual woman. The wrong prescription for women: How medicine and media create a “need” for treatments, drugs, and surgery.

[B84-ejihpe-16-00077] Vasconcelos P., Paúl C., Serruya S. J., Ponce de León R. G., Nobre P. (2023). A systematic review of sexual health and subjective well-being in older age groups. Revista Panamericana de Salud Pública.

[B85-ejihpe-16-00077] Viana H. B., de Guirardello E. B., Madruga V. A. (2010). Tradução e adaptação cultural da escala ASKAS—Aging sexual knowledge and attitudes scale em idosos brasileiros. Texto Contexto Enfermagem, Florianópolis.

[B86-ejihpe-16-00077] Viana H. B., Madruga V. A., Guirardello E. B., Silva D. (2012). Adaptação e validação da ASKAS—Aging Sexual Knowledge and Attitudes Scale em idosos brasileiros. Revista Kairós Gerontologia.

[B87-ejihpe-16-00077] Villar F., Celdrán M., Fabà J., Serrat R. (2014). Barriers to sexual expression in residential aged care facilities (RACFs): Comparison of staff and residents’ views. The Journal of Advanced Nursing.

[B88-ejihpe-16-00077] Von Humboldt S., Leal I., Low G. (2025a). Sexuality, love, and sexual well-being in old age. International handbook of love: Transcultural and transdisciplinary perspectives.

[B89-ejihpe-16-00077] von Humboldt S., Ribeiro-Gonçalves J. A., Low G., Leal I. (2025b). What sexual well-being really means for older adults: A systematic review of the literature. Educational Gerontology.

[B90-ejihpe-16-00077] Waite L. J., Laumann E. O., Das A., Schumm L. P. (2009). Sexuality: Measures of partnerships, practices, attitudes, and problems in the national social life, health, and aging study. Journal of Gerontology: Social Sciences.

[B91-ejihpe-16-00077] Wang T.-F., Lu C.-H., Chen I.-J., Yu S. (2008). Sexual knowledge, attitudes and activity of older people in Taipei, Taiwan. Journal of Clinical Nursing.

[B92-ejihpe-16-00077] White C. B. (1982). A scale for the assessment of attitudes and knowledge regarding sexuality in the aged. Archives of Sexual Behavior.

[B93-ejihpe-16-00077] White C. B. (1998). Aging sexual knowledge and attitudes scale. Handbook of sexuality-related measures.

[B94-ejihpe-16-00077] World Health Organization (2006). Sexual health document series. Defining sexual health: Report of technical consultation on sexual health 28–31 January 2002, Geneva.

[B95-ejihpe-16-00077] World Health Organization (2020). UN decade of healthy ageing: Plan of action 2021–2030.

[B96-ejihpe-16-00077] World Health Organization (2023). Sexual and reproductive health and research (SRH).

[B97-ejihpe-16-00077] Yan E., Lee C. F. (2013). Chinese version of Aging Sexual Knowledge and Attitudes Scale. Asian Journal of Gerontology & Geriatrics.

[B98-ejihpe-16-00077] Zhang F., Yang Z., Li X., Wang A. (2023). Factors influencing the quality of sexual life in the older adults: A scoping review. International Journal of Nursing Sciences.

