# Supplementary material for: Sexual Knowledge, Attitudes, and Motivational Factors Associated with Sexual Activity in Older Adults: A Cross-Sectional Study
Source: Eur J Investig Health Psychol Educ. 2026 May 30;16(6):77. doi: 10.3390/ejihpe16060077 (PMC13298553; doi:10.3390/ejihpe16060077)
Supplement: Supplementary file 1 [file ejihpe-16-00077-s001.zip › ejihpe-4269182-supplementary.pdf]

Supplementary Table S1. Percentage of correct and incorrect responses to knowledge items on sexuality in older adulthood.

Item	Correct (%)	Incorrect (%)
Males over the age of 65 typically take longer to attain an erection than younger males.	78.0	22.0
Males over the age of 65 usually experience a reduction in intensity of orgasm relative to younger males.	59.7	40.3
The firmness of erection in older males is often less than that of younger males.	79.5	20.5
Older females (65 years and older) have reduced vaginal lubrication compared to younger females.	77.3	22.7
Older females take longer to achieve adequate vaginal lubrication than younger females.	79.5	20.5
Older females may experience painful intercourse due to reduced vaginal elasticity and decreased lubrication.	62.2	37.8
An important factor in maintaining sexual responsiveness in aging males is the consistency of sexual activity throughout life.	51.1	48.9
There is an inevitable loss of sexual satisfaction in postmenopausal women.	35.5	64.5
Older men are more likely than older women to have a stronger interest in maintaining an active sexual life.	21.5	78.5
Sexual activity declines in older age due to loss of physical attractiveness in men and women.	48.4	51.6
Health problems are more important than sexual problems in later life.	85.5	14.5
Older men and women do not find each other attractive.	40.9	59.1
Sexually active older adults do not need to use condoms.	59.1	40.9
Some medications may reduce sexual desire and performance in both men and women.	75.3	24.7
Chronic conditions such as diabetes or hypertension prevent older adults from maintaining a fulfilling sexual life.	26.9	73.1
Sexuality is typically a lifelong need.	72.6	27.4
There is evidence that sexual activity in older adults has beneficial physical effects.	57.8	42.2

Note. Percentages represent the proportion of correct and incorrect responses for each item.

Supplementary Table S2. Percentage distribution of responses to attitude items on sexuality in older adulthood.

Item	SD (%)	D (%)	N (%)	A (%)	SA (%)
Older adults (65 years and older) have little interest in sexuality.	11.4	19.0	27.7	29.3	12.5
Institutions such as nursing homes should encourage and support sexual activity among their residents.	22.4	21.3	27.3	18.6	10.4
If a relative of mine, living in a nursing home, was to have a sexual relationship with another resident, I would complain to the management.	46.2	23.9	19.0	5.4	5.4

I would like to know more about the changes in sexual functioning in older years.	6.0	16.3	24.5	31.0	22.3
I would support sex education courses for aged residents of nursing homes.	8.2	9.8	25.0	27.7	29.3
I would support sex education courses for the staff of nursing homes.	8.2	6.5	21.7	28.3	35.3
Masturbation is an acceptable sexual activity for older males.	7.1	8.7	22.3	25.0	37.0
Masturbation is an acceptable sexual activity for older females.	8.2	10.9	23.4	21.7	35.9
Institutions, such as nursing homes, should provide privacy so as to allow residents to engage in sexual behavior without fear of intrusion or observation.	7.1	5.4	17.9	31.5	38.0
It is appropriate for a nursing home to prohibit a widowed resident from engaging in a relationship if the family does not approve.	39.1	29.3	13.6	13.0	4.9
The sexual rights and freedoms of older adults living in nursing homes should be protected.	2.2	3.3	14.7	27.2	52.7
Older adults living in nursing homes should be allowed to invite an external partner to engage in sexual activity.	15.2	14.7	21.7	28.3	20.1
Older adults should be allowed to possess pornographic material.	25.0	19.0	28.8	14.1	13.0
Two older residents should not be allowed to spend the night together if they are not in a stable relationship.	35.9	27.7	17.4	11.4	7.6
Sexual relationships between two older women should be prohibited.	46.8	19.4	17.2	7.5	9.1
Sexual relationships between two older men should be prohibited.	43.5	21.0	18.8	7.0	9.7
Older adults should be encouraged to remain sexually active.	8.1	10.2	35.5	24.7	21.5
Sexuality should only be expressed by young people.	52.7	22.0	10.2	12.4	2.7
Older adults lose interest in sexual relationships after widowhood.	13.6	18.5	23.9	26.6	17.4

Note. SD = Strongly Disagree; D = Disagree; N = Neutral; A = Agree; SA = Strongly Agree.
